# Decrease in fasting insulin secretory function correlates with significant liver fibrosis in Japanese non‐alcoholic fatty liver disease patients

**DOI:** 10.1002/jgh3.12367

**Published:** 2020-06-09

**Authors:** Norimasa Araki, Hirokazu Takahashi, Ayako Takamori, Yoichiro Kitajima, Hideyuki Hyogo, Yoshio Sumida, Saiyu Tanaka, Keizo Anzai, Shinichi Aishima, Kazuaki Chayama, Kazuma Fujimoto, Yuichiro Eguchi

**Affiliations:** ^1^ Department of Internal Medicine, Faculty of Medicine Saga University Saga Japan; ^2^ Clinical Research Center Saga University Hospital Saga Japan; ^3^ Liver Center Saga University Hospital Saga Japan; ^4^ Department of Gastroenterology and Hepatology JA Hiroshima General Hospital Hatsukaichi Japan; ^5^ Division of Hepatology and Pancreatology, Department of Internal Medicine Aichi Medical University Aichi Japan; ^6^ Center for Digestive and Liver Disease Nara City Hospital Nara Japan; ^7^ Department of Pathology & Microbiology, Faculty of Medicine Saga University Saga Japan; ^8^ Department of Gastroenterology and Metabolism Graduate School of Biomedical and Health Sciences, Hiroshima University Hiroshima Japan; ^9^ Faculty of Medicine International University of Health and Welfare Fukuoka Japan

**Keywords:** homeostatic model assessment‐beta cell function, non‐alcoholic fatty liver disease, obesity, pancreatic β‐cell function

## Abstract

**Background and Aim:**

Non‐alcoholic fatty liver disease (NAFLD) is typically associated with metabolic syndrome and diabetes, and insulin resistance is involved in its pathogenesis. However, the relationship between insulin secretion and NAFLD is unclear. We aimed to characterize the relationship between fasting insulin secretory function (ISF), evaluated using the homeostatic model assessment‐beta cell function (HOMA‐β) and the severity of fibrosis during NAFLD.

**Methods:**

A‐β was calculated in 188 patients with biopsy‐confirmed NAFLD, and the correlations between Log HOMA‐β and clinical parameters, including hepatic fibrosis, were calculated.

**Results:**

Log HOMA‐β was significantly lower in NAFLD patients with significant fibrosis (stages 2–4) than in those in the early stages (stages 0–1) (median [interquartile range]) (2.1 [1.9–2.4] *vs* 2.0 [1.8–2.2], *P* = 0.04). The prevalence of significant fibrosis decreased with increasing Log HOMA‐β: it was 59.2% in participants with low ISF (Log HOMA‐β < 1.85), 43.6% in those with intermediate ISF (1.85 ≤ Log HOMA‐β < 2.25), and 68.0% in those with high ISF (Log HOMA‐β ≥ 2.25). Patients with lower Log HOMA‐β had lower current body mass index (BMI), BMI at 20 years of age, and peak lifetime BMI than patients with intermediate or high Log HOMA‐β.

**Conclusions:**

Fasting ISF decreased alongside the development of liver fibrosis in NAFLD, suggesting that an impaired β cell function has a characteristic finding of significant liver fibrosis in relatively nonobese Japanese patients.

## Graphical abstract

Japanese patients with NAFLD have significant fibrosis with reduced insulin secretion. These patients also tended to be significantly non‐obese.

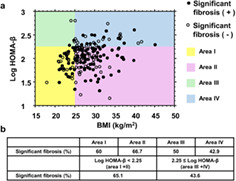

## Introduction

Non‐alcoholic fatty liver disease (NAFLD) is a manifestation of metabolic syndrome in the liver and is often associated with insulin resistance, hyperglycemia, and diabetes mellitus.^1^, ^2^, ^3^ The liver fibrosis that occurs during NAFLD is a risk for hepatocarcinogenesis, cardiovascular disease, and poor disease prognosis,^4^, ^5^, ^6^ and several studies have demonstrated that insulin resistance exacerbates liver fibrosis in NAFLD.^7^, ^8^ Homeostasis model assessment‐insulin resistance (HOMA‐IR) has been shown to be useful for the early detection of NAFLD and liver fibrosis,^9^ but few studies have investigated the influence of insulin secretory function (ISF) on liver fibrosis in NAFLD.

The most common methods of evaluating fibrosis in NAFLD are the pathologic evaluation of a liver biopsy and/or noninvasive tests, including the fibrosis‐4 (FIB‐4) index.^10^ Insulin secretary dysfunction is reflected in impaired glucose tolerance, which precedes the development of diabetes mellitus, and in which β‐cells can no longer rapidly respond to changes in blood glucose concentration and adjust insulin secretion to compensate for systemic insulin resistance.^11^, ^12^ ISF was determined using homeostatic model assessment‐beta cell function (HOMA‐β), which is a conventional, noninvasive method of quantifying ISF.^13^, ^14^, ^15^ The principal aim of the present study was to characterize the relationship between ISF and the stage of progression of NAFLD evaluated by liver biopsy.

## Method

### *Patients*

A total of 208 patients who had been histologically diagnosed with NAFLD at Eguchi Hospital, Saga Medical School, Hiroshima University Hospital or Nara City Hospital between January 2004 and April 2010 were enrolled in the present study, but a further 20 patients for whom fasting insulin or glucose data were not available or who had been treated with insulin, a sulfonylurea, and/or a glinide were excluded. Finally, 188 patients were included in this study. All the participants were negative for hepatitis B virus surface antigen (HBs‐Ag) and/or anti‐hepatitis C virus antibody (HCV‐Ab). Habitual alcohol drinkers (men > 30 g/day and women > 20 g/day) and patients who had been diagnosed with another liver disease, such as autoimmune hepatitis, primary biliary cholangitis, primary sclerosing cholangitis, or malignancy, were excluded from the present analysis. Informed consent was obtained from all the participants in the form of an opt‐out on the website. The study was approved by the relevant institutional review board at each institution and was conducted in accordance with the principles of the Declaration of Helsinki.

### *Physical examination and serum biochemistry*

**Figure 1:**
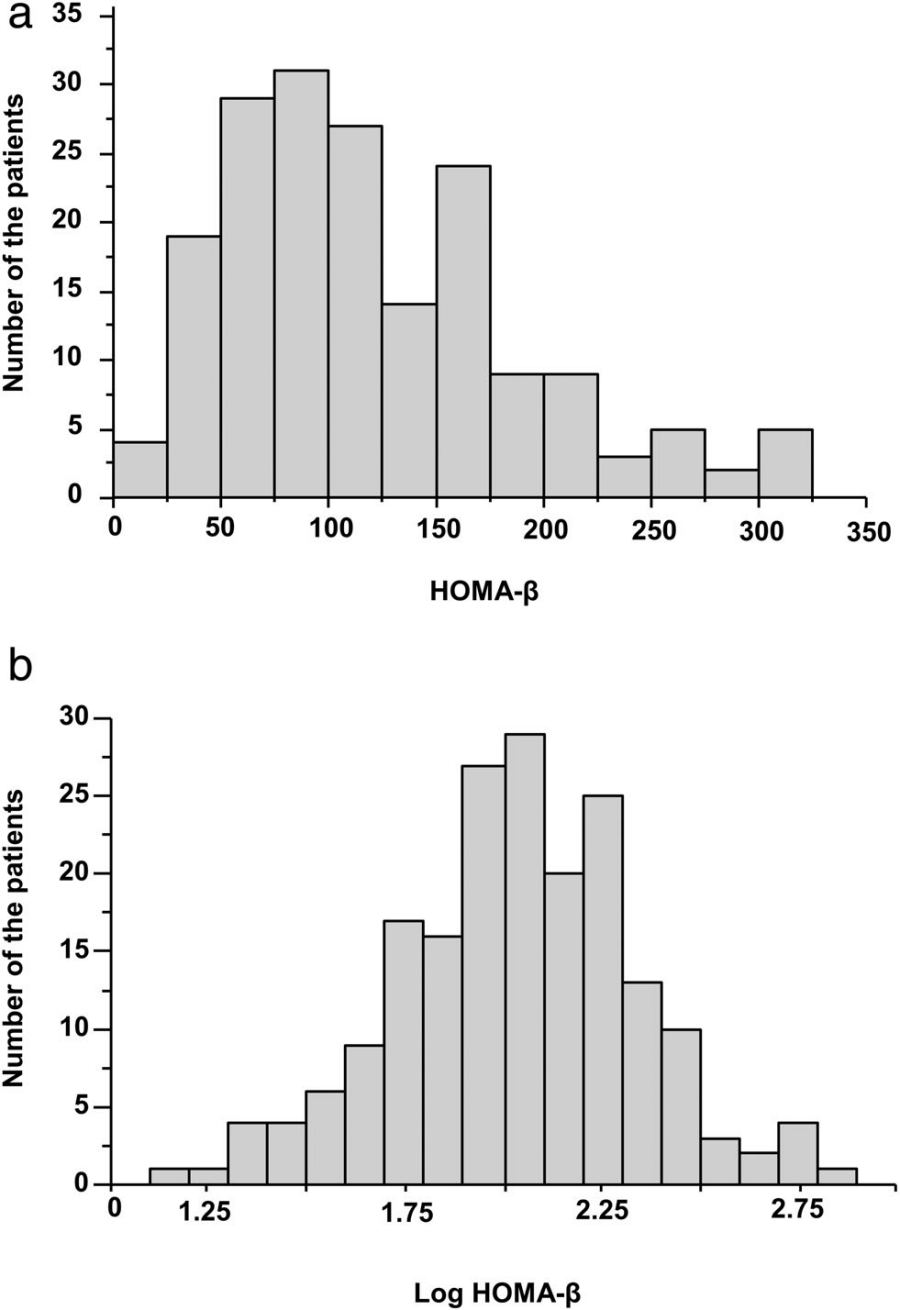
Distribution of participants with non‐alcoholic fatty liver disease according to homeostasis model assessment for β cell function (HOMA‐β) and Log HOMA‐β. (a) The HOMA‐β values of the participants were not normally distributed. (b) The Log HOMA‐β values were normally distributed.

Body mass and height were measured for the calculation of body mass index (BMI). Venous blood samples were taken from all the participants following a 12‐h overnight fast, and HbA1c, aspartate aminotransferase (AST), alanine aminotransferase (ALT), γ‐glutamyl transferase (GGT), total cholesterol (TC), high‐density lipoprotein‐cholesterol (HDLC), low‐density lipoprotein‐cholesterol (LDLC), triglycerides (TG), fasting insulin, fasting plasma glucose (FPG), and immunoreactive insulin (IRI) were measured using standard techniques and commercially available kits. Visceral fat area (VFA) was measured by computed tomographic scan. FIB‐4 index was calculated as (age [years] × AST [U/L])/(platelet count [10^9^/μL]) × √ ALT [U/L]. Basal insulin secretion and insulin sensitivity were evaluated using homeostasis models, as previously published.^13^ HOMA‐β was calculated using the basal glucose and insulin concentrations: fasting plasma insulin [μU/mL] × 360/fasting plasma glucose [mg/dL] − 63, and used to evaluate pancreatic β‐cell insulin secretion under unstimulated conditions. The HOMA‐β values were not normally distributed (Fig. 1a), but the logarithmically transformed (Log HOMA‐β) data were normally distributed, as shown in Figure 1b, and Log HOMA‐β was used in all the analyses of this study. The participants were classified into three groups according to their interquartile of Log HOMA‐β: (i) a low ISF group [Log HOMA‐β < 1.85 (< first percentile)], (ii) an intermediate ISF group [1.85 ≤ Log HOMA‐β < 2.25 (first–third percentile)], and (iii) a high ISF group [Log HOMA‐β ≥ 2.25 (≥ third percentile)]. HOMA‐IR was calculated as fasting insulin [μU/mL] × fasting glucose [mg/dL]/405.^16^

### *Pathology*

Percutaneous liver biopsy samples had been obtained from the participants under ultrasonic guidance. Formalin‐fixed, paraffin‐embedded liver sections were stained with hematoxylin and eosin, silver reticulin, or Azan and were evaluated by an experienced pathologist (S.A.). Histological diagnosis of NAFLD was performed if hepatic steatosis was 5% or more according to Kleiner *et al*.^17^ In the current study, four cirrhotic cases without steatosis were included according to the clinical and pathological history of NAFLD and considered to be the “burn‐out NASH.” Grading and staging were performed according to Brunt *et al*.^18^ and Kleiner *et al*.,^17^ and NAFLD activity scores (NAS) were assigned as previously reported.^19^ According to the pathologic findings and Kleiner's classification, NAFLD patients with stage 0 or 1 liver fibrosis were defined as having early NAFLD, and those with stages ≥2 (severe) liver fibrosis were defined as having NAFLD with significant fibrosis. NASH and non‐alcoholic fatty liver (NAFL) were diagnosed according to the fatty liver inhibition of progression (FLIP) algorithm.^20^

### *Statistical analysis*

Means and standard deviations were calculated for all continuous variables, and the Mann–Whitney *U*‐test and Kruskal‐Wallis test ± a *post‐hoc* test were performed for nonparametric data. Pearson's chi‐square test was used for the analysis of categorical parameters. Multivariate logistic regression was performed to identify the factors associated with significant liver fibrosis (stage ≥ 2). The explanatory variables in the multivariate analysis were those that were significant in the univariate analyses. Differences were considered significant when *P* < 0.05. All analyses were performed using SPSS Statistics version 21 (IBM, Armonk, NY, USA).

## Results

### *Relationships between* *ISF* *and clinical characteristics*

The clinical characteristics of the participants were summarized and compared according to the ISF category (Table 1). There were no gender differences among the groups. The median BMI of the whole group of participants was 27.8 kg/m^2^, but that of 154 of the 188 participants (81.9%) was >25 kg/m^2^, representing relative obesity, according to the Japanese criteria^21^, ^22^, ^23^. BMI significantly increased with ISF. The median AST, ALT, and GGT activities of the participants as a whole were outside their normal ranges. The low ISF group had normal liver enzymes and metabolic parameters, including, ALT, GGT, TG, BMI, and VFA, in contrast to the intermediate and high ISF groups. Across the participants as a whole, median FPG, insulin, HOMA‐IR, HOMA‐β, and Log HOMA‐β were above the normal range, indicating the presence of prediabetes and insulin resistance. These parameters increased with ISF. HbA1c, TC, LDL, and ferritin were not related to ISF. TG and VFA increased with the severity of ISF, whereas HDLC was inversely related to ISF. The FIB‐4 index was significantly higher in the low ISF group than in the other groups. The participants with significant fibrosis were more likely to be in the low ISF (59.2%) and intermediate ISF (68.0%) groups than the high ISF group (43.6%). On the other hand, NASH was diagnosed more frequently in the intermediate ISF (87.0%) and high ISF (82.1%) than low ISF (69.4%).

**Table 1:** Participant characteristics, categorized according to insulin secretory function (ISF)

	Overall (*n* = 188)	Low ISF (*n* = 49)	Intermediate ISF (*n* = 100)	High ISF (*n* = 39)	*P*‐value
Female (%)	45.7	46.9	51.0	30.8	0.07
Age (years)	52.0 (41.0–64.0)	59.0 (46.3–63.8)	53.5 (45.0–65.0)	37.0 (30.3–54.8)	<0.001
BMI (kg/m^2^)	27.8 (25.4–31.2)	26.6 (24.1–29.5)	27.4 (25.5–30.1)	31.9 (27.9–36.2)	<0.001
PLT (×10^4^/μL)	22.0 (18.5–26.2)	21.0 (17.6–25.2)	22.5 (18.3–26.2)	22.2 (18.7–26.9)	0.51
AST (U/L)	42.0 (31.0–58.2)	42.0 (26.3–62.0)	42.0 (31.0–57.0)	45.0 (38.0–59.8)	0.39
ALT (U/L)	67.0 (43.0–93.0)	51.0 (32.3–95.8)	62.0 (48.0–82.0)	78.0 (56.8–138.8)	0.049
GGT (U/L)	66.0 (41.0–105.2)	61.0 (31.0–120.3)	61.0 (35.0–96.0)	87.0 (58.0–134.3)	0.02
FPG (mg/dL)	109.0 (95.0–125.0)	126.0 (101.3–154.0)	95.0 (105.5–117.0)	93.0 (83.3–103.0)	<0.001
IRI (μU/mL)	14.3 (10.5–20.7)	9.0 (5.2–12.2)	14.4 (10.6–18.9)	25.7 (16.4–32.9)	<0.001
HbA1c (%)	5.9 (5.4–6.4)	5.9 (5.5–6.7)	5.9 (5.8–6.2)	5.4 (5.1–6.0)	0.20
HOMA‐IR	4.0 (2.5–6.2)	2.5 (1.4–5.2)	3.9 (2.6–5.6)	6.0 (3.5–8.8)	0.003
HOMA‐β	110.3 (70.4–168.3)	50.9 (37.3–60.0)	111.6 (92.4–149.1)	248.4 (203.3–302.9	<0.001
Log HOMA‐β	2.0 (1.9–2.2)	1.7 (1.6–1.8)	2.1 (2.0–2.2)	2.4 (2.3–2.5)	<0.001
TC (mg/dL)	201.5 (176.0–233.0)	201.0 (174.3–233.5)	199.0 (176.8–227.3)	208.0 (177.3–241.0)	0.82
HDLC (mg/dL)	48.0 (42.0–58.0)	50.0 (43.0–62.8)	50.0 (43.0–58.5)	44.0 (38.0–50.5)	0.01
LDLC (mg/dL)	127.0 (104.5–145.5)	126.5 (90.0–148.0)	127.0 (104.0–144.0)	127.0 (110.3–146.0)	0.90
TG (mg/dL)	147.0 (102.2–215.3)	129.0 (101.8–193.0)	142.0 (101.0–200.0)	196.0 (118.0–307.5)	0.02
Ferritin (ng/mL)	176.2 (100.3–262.6)	205.2 (84.3–289.9)	161.6 (101.2–244.0)	220.9 (124.5–322.5)	0.37
VFA (cm^2^)	145.5 (112.6–176.2)	133.2 (112.4–171.2)	144.2 (114.0–175.5)	167.5 (134.1–233.2)	0.03
FIB‐4 index	1.2 (0.7–1.8)	1.4 (1.0–1.9)	1.3 (0.9–1.9)	0.9 (0.6–1.4)	0.005
NAS steatosis (0/1/2/3)	5/63/72/48	3/18/17/11	2/41/37/20	0/4/18/17	0.003
NAS Inflammation (0/1/2/3)	29/99/46/14	12/25/9/3	12/55/24/9	5/19/13/2	0.41
NAS ballooning (0/1/2)	25/66/97	10/19/20	9/38/53	6/9/24	0.04
Fibrosis (0/1/2/3/4)	36/38/57/53/4	11/9/16/9/4	17/15/32/36/0	8/14/9/8/0	0.005
Early/Significant fibrosis	74/114	20/29	32/68	22/17	0.04
NAFL/NASH	35/153	15/34	13/87	7/32	0.08

The median values and ranges are shown. *P*‐values were obtained using the Kruskal‐Wallis test. Low ISF: Log HOMA‐β <1.85, Intermediate ISF: 1.85 ≤ Log HOMA‐β <2.25, and High ISF: Log HOMA‐β ≥ 2.25. BMI: body mass index. PLT: platelet count. AST: aspartate transaminase.

ALT, alanine aminotransferase; FPG, fasting plasma glucose; GGT, γ‐glutamyl transferase; HbA1c, hemoglobin A1c; HDLC, high‐density lipoprotein‐cholesterol; HOMA‐IR, homeostasis model assessment‐insulin resistance; HOMA‐β, homeostasis model assessment‐beta cell function; IRI, immunoreactive insulin; LDLC, low‐density lipoprotein‐cholesterol; NAFL, non‐alcoholic fatty liver; NASH, non‐alcoholic steatohepatitis; TC, total cholesterol; TG, triglycerides; VFA, visceral fat area.

### *Characteristics of participants with early* *NAFLD* *or significant fibrosis* *NAFLD*

Patient characteristics are summarized according to the degree of progression of NAFLD fibrosis in Table 2. The participants with early NAFLD were significantly younger than those with significant fibrosis. There were no significant differences in BMI, PLT, ALT, IRI, HbA1c, ferritin, and VFA between the early NAFLD and NAFLD with significant fibrosis, whereas AST, GGT, and FIB‐4 index were significantly higher in the significant fibrosis group. TG and HDLC did not differ among the groups, but they were significantly lower in the significant fibrosis group.

**Table 2:** Participant characteristics, categorized according to the severity of fibrosis during non‐alcoholic fatty liver disease (NAFLD)

	Early NAFLD (*n* = 74)	Significant fibrosis NAFLD (*n* = 114)	*P* value
Female (%)	40.5	49.1	0.63
Age (years)	52.0 (42.0–57.5)	54.5 (41.0–65.0)	0.04
BMI (kg/m^2^)	26.5 (25.5–30.3)	28.6 (25.2–31.4)	0.72
PLT (×10^4^/μL)	23.0 (20.9–26.8)	21.6 (17.4–26.1)	0.07
AST (U/L)	37.5 (25.5–52.0)	45.0 (32.8–59.0)	0.02
ALT (U/L)	63.5 (32.5–94.5)	67.0 (45.8–93.0)	0.22
GGT (U/L)	60.5 (30.5–93.0)	66.0 (46.8–106.0)	0.02
FPG (mg/dL)	97.0 (92.0–109.5)	111.0 (97.5–130.5)	0.01
IRI (μU/mL)	13.1 (8.2–20.0)	14.9 (10.6–20.7)	0.24
HbA1c (%)	5.2 (5.8–6.4)	5.9 (5.6–6.5)	0.36
HOMA‐IR	3.2 (2.0–4.9)	4.3 (2.7–6.2)	0.04
HOMA‐β	111.6 (65.8–217.0)	109.5 (72.6–163.6)	0.20
Log HOMA‐β	2.1 (1.9–2.4)	2.0 (1.8–2.2)	0.04
TC (mg/dL)	213.0 (188.3–241.0)	199.0 (174.0–228.8)	0.02
HDLC (mg/dL)	48.0 (39.0–57.0)	48.5 (42.0–59.0)	0.22
LDLC (mg/dL)	143.5 (129.5–159.0)	126.0 (98.5–143.5)	<0.001
TG (mg/dL)	166.0 (118.5–222.5)	144.0 (102.0–211.8)	0.3
Ferritin (ng/mL)	219.4 (112.5–303.7)	171.5 (98.7–252.4)	0.28
VFA (cm^2^)	132.3 (102.3–168.7)	151.2 (120.7–183.1)	0.24
FIB‐4 index	0.97 (0.61–1.48)	1.34 (0.91–2.13)	0.03

Median values and ranges are shown. *P*‐values were obtained using the Mann–Whitney U‐test.

ALT, alanine aminotransferase; AST, aspartate transaminase; BMI, body mass index; FPG, fasting plasma glucose; GGT, γ‐glutamyl transferase; HbA1c, hemoglobin a1c; HDLC, high‐density lipoprotein‐cholesterol; HOMA‐IR, homeostasis model assessment‐insulin resistance; HOMA‐β, homeostasis model assessment‐beta cell function; IRI, immunoreactive insulin; LDLC, low‐density lipoprotein‐cholesterol; PLT, platelet count; TC, total cholesterol; TG, triglycerides; VFA, visceral fat area.

**Figure 2:**
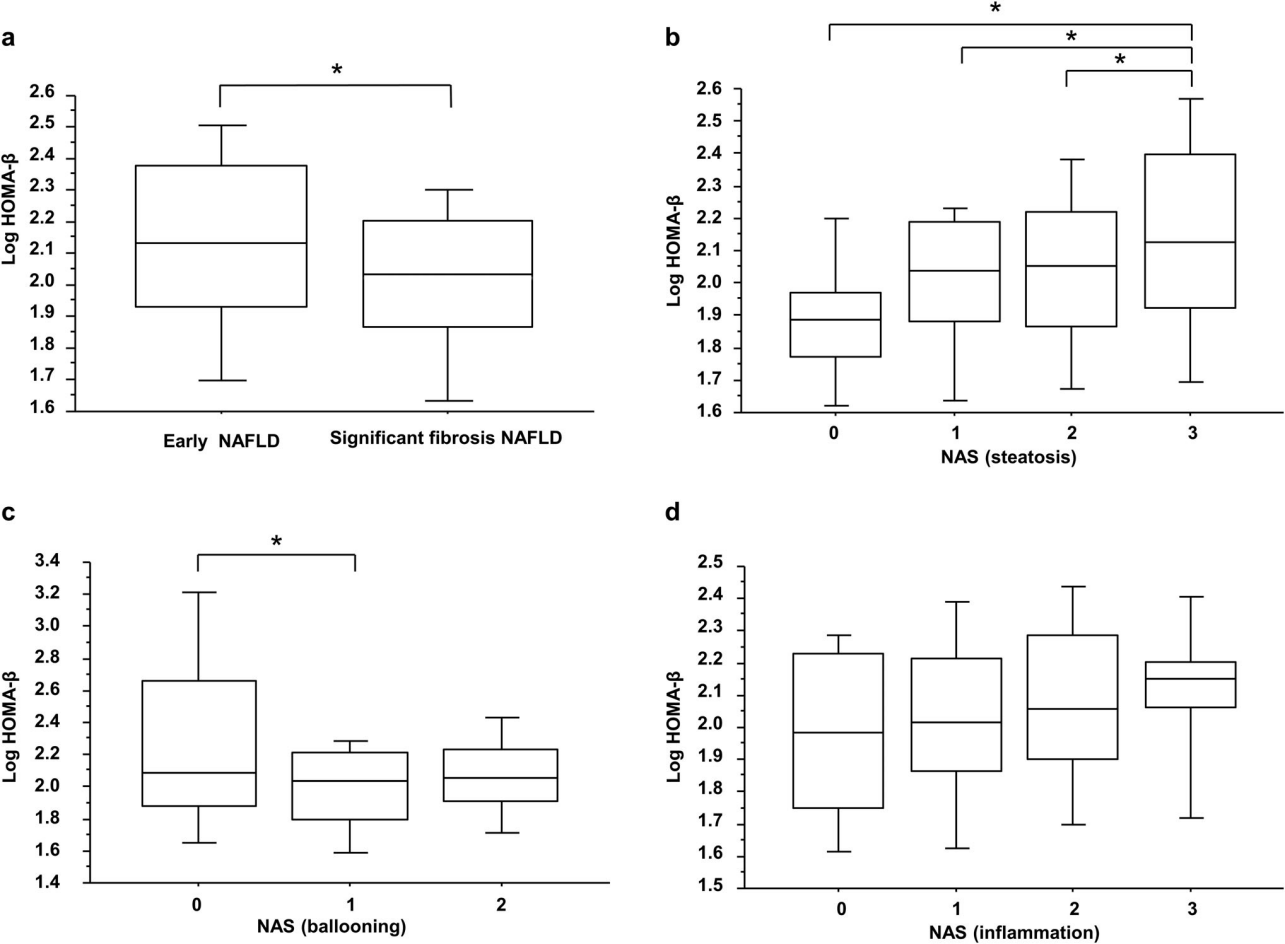
Relationship between log HOMA‐β (homeostasis model assessment‐beta cell function) and pathologic evaluation of non‐alcoholic fatty liver disease. (a) Staging of liver fibrosis: early fibrosis and significant fibrosis. (b) NAFLD activity score (NAS) for steatosis. (c) NAS for ballooning. d: NAS for inflammation. *P < 0.05.

The HOMA‐IR, HOMA‐β, and Log HOMA‐β data are shown in Table 2 and Figure 2. HOMA‐IR increased with the progression of fibrosis, whereas Log HOMA‐β decreased with the progression of fibrosis, as shown in Figure 2a. Figure 2b–d show the relationships between Log HOMA‐β and histopathologic findings in the livers of the participants. The severity of steatosis increased and that of hepatocyte ballooning decreased as Log HOMA‐β increased, but the severity of inflammation was not related to Log HOMA‐β. Median (range) of Log HOMA‐β was 1.97 (1.80–2.10) in NAFL and 2.05 (1.90–2.22) in NASH, and there was no significant difference (p = 0.13).

### *Association among significant fibrosis, Log* *HOMA‐β, and* *BMI*

**Figure 3:**
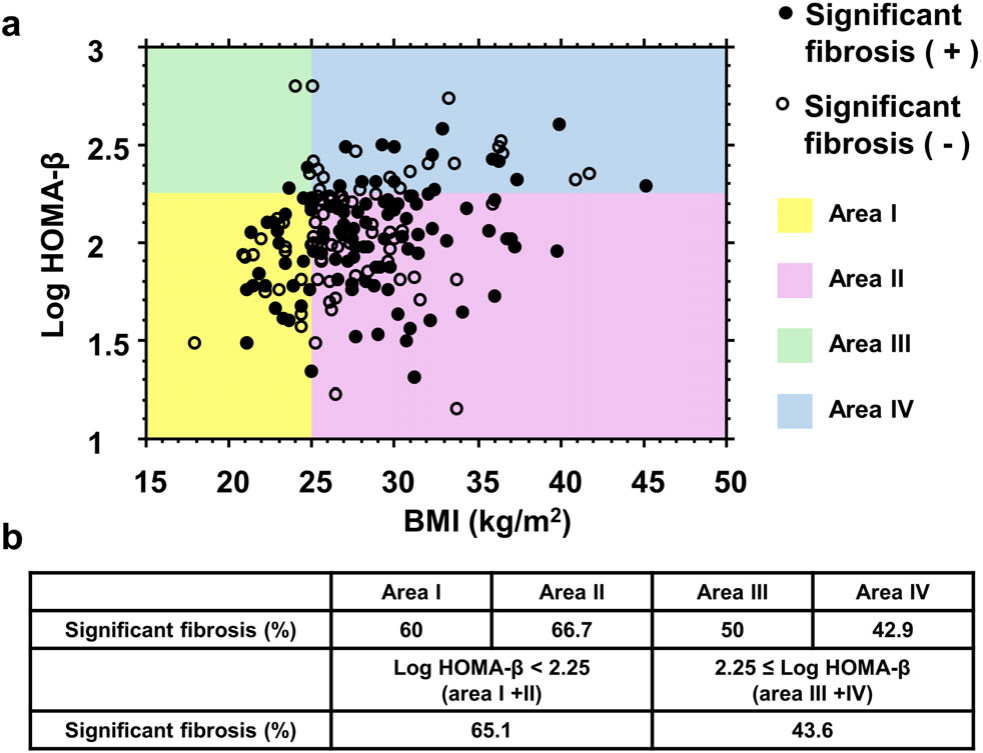
Association among significant fibrosis, Log HOMA‐β, and BMI. (a) Correlation diagram was divided into four areas according to Log HOMA‐β and BMI. Area I, Log HOMA‐β < 2.25 and BMI < 25; Area II, Log HOMA‐β < 2.25 and BMI ≥ 25; Area III, Log HOMA‐β ≥ 2.25 and BMI < 25; Area IV, Log HOMA‐β ≥ 2.25 and BMI ≥ 25. Black dots represent the patients with significant fibrosis, and open dots represent those without significant fibrosis. (b) Prevalence of the patients with significant fibrosis in the individual areas.

Figure 3 shows correlation between Log HOMA‐β and BMI, and prevalence of significant fibrosis was assessed in the individual area divided by Log HOMA‐β and BMI. In area I (low Log HOMA‐β and low BMI) and area II (low Log HOMA‐β and high BMI), the prevalence of significant fibrosis was 60% and 66.7%, respectively, which was higher than area III (high Log HOMA‐β and low BMI: 50%) and area IV (high Log HOMA‐β and high BMI: 42.9%).

### *Characteristic associated with significant liver fibrosis in* *NAFLD*

The results of a multivariate analysis to identify the characteristics associated with significant fibrosis in NAFLD are shown in Table 3. Low PLT and high AST, GGT, FPG, and HOMA‐IR were the factors for significant liver fibrosis. Low ISF and intermediate ISF were independently associated with significant fibrosis in NAFLD.

**Table 3:** Factors associated with significant liver fibrosis in participants with non‐alcoholic fatty liver disease

	Odds ratio	*P* value	95% confidence interval
PLT ≤ 20 × 10^4^/μL	2.30	0.15	0.73–7.264
AST ≥ 25 U/L	5.77	0.02	1.38–24.17
GGT ≤ 50 U/L	3.67	0.01	1.46–9.20
TC ≥ 200 mg/dL	0.73	0.62	0.21–2.56
LDLC ≥ 140 mg/dL	0.22	0.12	0.11–1.29
Low or intermediate ISF (Log HOMA‐β ≤ 2.25)	2.82	0.03	1.12–7.10
FIB‐4 index ≥ 1.3	2.30	0.15	0.73–7.26

Gender and age were adjusted for in the logistic regression analysis.

AST, aspartate transaminase; FPG, fasting plasma glucose; GGT, γ‐glutamyl transferase; ISF, insulin secretory function; LDLC, low density lipoprotein‐cholesterol; PLT, platelet count; TC, total cholesterol.

### *Relationships between* *ISF* *and current and past* *BMI*

**Figure 4:**
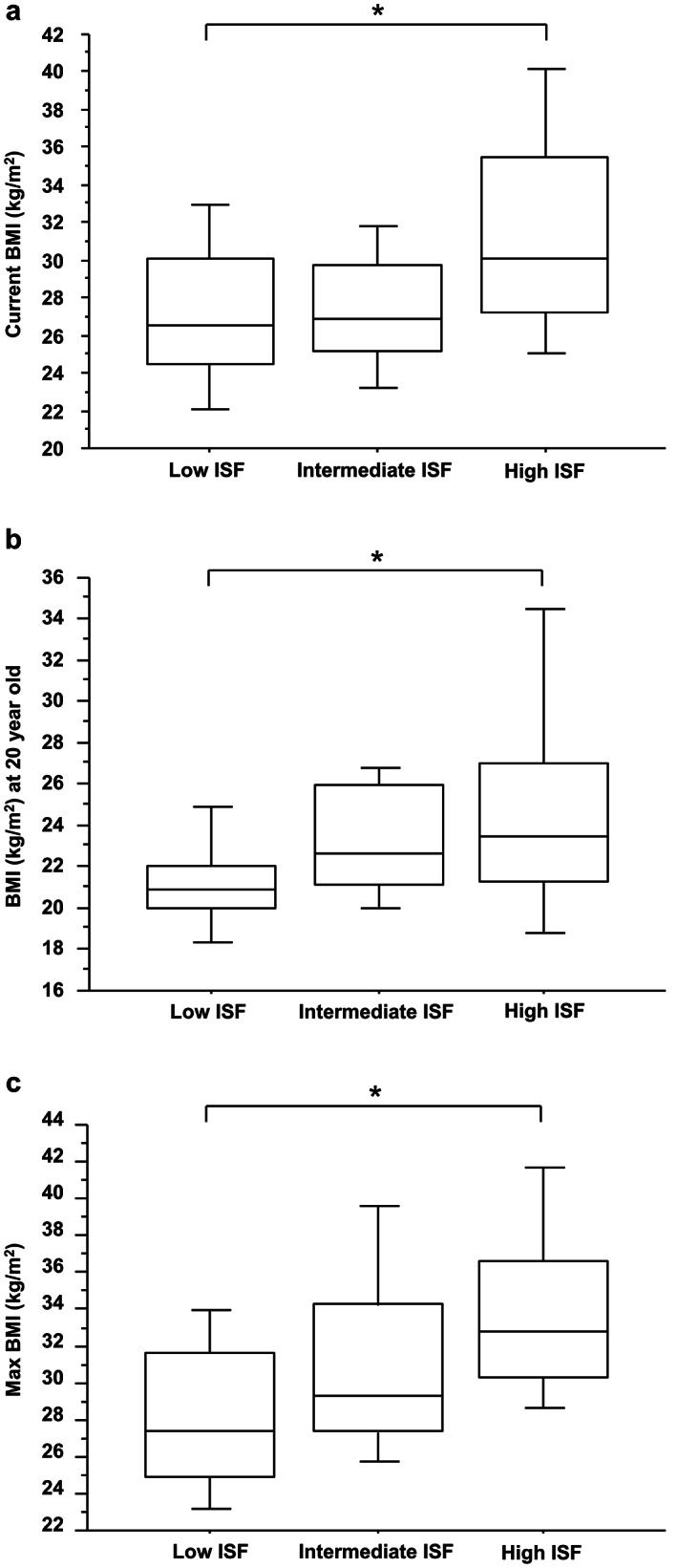
Relationship between body mass index (BMI) and insulin secretory function (ISF) in participants with non‐alcoholic fatty liver disease. (a) The present BMI of the participants. (b) The BMI of the participants when 20 years old. (c) The lifetime peak BMI of the participants. *P < 0.05.

As shown in Figure 4a, current BMI decreased with decreasing ISF in NAFLD patients, and the relationships between ISF and BMI at 20 years old (Fig. 4b) and the lifetime peak BMI (Fig. 4c) were also significant, implying positive correlations between BMI (past and current) and current ISF in the participants.

## Discussion

The present study shows that fasting ISF, evaluated using HOMA‐β, decreases with the severity of liver fibrosis in Japanese NAFLD patients, and the BMI of the participants, which was relatively low at the time of, and previous to, the study, was not a related factor for significant liver fibrosis. Few previous clinical studies have investigated the relationship between ISF and the pathogenesis of NAFLD. Although ISF, evaluated by glucose tolerance testing, has been found to be lower in NAFLD patients,^24^, ^25^, ^26^ it has been shown not to differ between NAFLD patients with fibrosis stages 0–1 and those with stages 2–3.^24^ However, the relationship with fasting ISF has not previously been evaluated.^24^, ^25^, ^26^ A study performed in Italy demonstrated that insulin secretion was impaired in the glucose tolerance test in Italian patients with NAFLD, but it was not observed in patients with simple steatosis.^27^ However, in this study, there was no detailed investigation regarding associations between histological findings and insulin secretion. In the present study, histological findings including liver fibrosis were evaluated and demonstrated significant correlation with ISF evaluated by Log HOMA‐β, suggesting that not only postchallenge insulin secretion in glucose tolerance test but also fasting ISF are associated with the development of NAFLD fibrosis. A study performed in the United States demonstrated that HOMA‐β, which was high in patients with either NASH or NAFLD, increased alongside the severity of fibrosis in NAFLD patients,^28^ which was in contrast to the findings of the present study. A possible explanation for this discrepancy is genetical or racial difference in the study cohort. It is well established that Asians have lower ISF and are less obese compared to Caucasians with diabetes.^29^ Indeed, in our study, patients with low ISF showed significantly lower current and past BMI than those with High ISF (Figs. 3 and 4). These results suggest that low ISF might contribute to avoiding obesity, while it might promote liver fibrosis in NAFLD. Therefore, we hypothesize that there might be a particular segment in Asian NAFLD, a disease type with lower ISF and less obesity, which is different from a common disease type in Caucasians concomitant with high ISF, insulin resistance, and obesity. Several studies, including ours, reported that the prevalence of “Lean NASH” with BMIs less than 25 kg/m^2^ is observed in around 10–20% of Asians, which is higher than western countries.^30^, ^31^, ^32^ Our findings might partly explain the high prevalence of lean NASH in Asia.

Recent experimental and clinical evidence suggests the existence of a β‐cell‐liver axis. Fibroblast growth factor‐21 (FGF21), which is expressed in fat, skeletal muscle, and liver, increases glucose‐induced ISF in rodent islets,^33^ and the plasma FGF21 increases with the severity of liver fibrosis in NAFLD.^34^ Clinical trials of FGF21 administration to NAFLD patients showed improvements in liver steatosis and fibrosis.^35^, ^36^ In addition, the concentration of circulating betatrophin, which increases in NAFLD patients according to the extent of their hepatic steatosis,^37^, ^38^ was higher in patients with liver fibrosis than in healthy controls.^39^ These reports suggest an association between the pathogenesis of NAFLD and circulating factors that might explain the observed relationship between ISF and liver fibrosis.

A major limitation of the present study was that we used HOMA‐β to evaluate ISF, which is affected by obesity‐ and age‐related insulin resistance,^40^, ^41^ such that ISF and insulin sensitivity are not clearly distinguished using this measure. Further evaluations of ISF using C‐peptide, hyperinsulinemic–euglycemic clamp and glucose tolerance testing should be undertaken in cross‐sectional and/or longitudinal studies to determine the nature of any causal relationships between ISF and liver fibrosis in NAFLD.

In conclusion, low ISF, evaluated using HOMA‐β, is a characteristic finding of significant liver fibrosis in Japanese NAFLD. Insulin secretion, as well as insulin resistance, can be evaluated to identify the potential risk of liver fibrosis progression.

## Author contribution

Norimasa Araki is the guarantor of the article. Norimasa Araki, Yoichiro Kitajima, and Ayako Takamori contributed to conceptualization and data analysis. Norimasa Araki, Hirokazu Takahashi, and Kazuma Fujimoto contributed to manuscript preparation. Norimasa Araki, Yoichiro Kitajima, Hideyuki Hyogo, Yoshio Sumida, Saiyu Tanaka, Shinichi Aishima, and Kazuaki Chayama contributed to data collection. Keizo Anzai, Kazuma Fujimoto, and Yuichiro Eguchi contributed to critical review of the manuscript.

## Declaration of conflict of interest

This study has not received any research grants. The authors declare that they have no conflict of interest.
